# Supplementary material for: The SERCA residue Glu340 mediates interdomain communication that guides Ca2+ transport
Source: Proc Natl Acad Sci U S A. 2020 Nov 23;117(49):31114–22. doi: 10.1073/pnas.2014896117 (PMC7733806; doi:10.1073/pnas.2014896117)
Supplement: Supplementary File [file pnas.2014896117.sapp.pdf]

Supplementary Information for

The SERCA residue Glu340 mediates inter-domain communication that guides Ca^{2+} transport

Maxwell M. G. Geurts[a,1], Johannes D. Clausen[b,c,1], Bertrand Arnou[b,d1], Cédric Montigny[d], Guillaume Lenoir[d], Robin A. Corey[a], Christine Jaxel[d], Jesper V. Møller[b], Poul Nissen[c,e], Jens Peter Andersen[b], Marc le Maire[d], and Maike Bublitz[a,2]

[a]Department of Biochemistry, University of Oxford, Oxford OX1 3QU, United Kingdom;
[b]Department of Biomedicine, Aarhus University, 8000 Aarhus C, Denmark;
[c]Department of Molecular Biology and Genetics, Aarhus University, 8000 Aarhus C, Denmark;
[d]Institute for Integrative Biology of the Cell (I2BC), Commissariat à l'Energie Atomique et aux Energies Alternatives, CNRS, Université Paris-Saclay, 91198 Gif-sur-Yvette, France;
[e]Danish Research Institute of Translational Neuroscience – DANDRITE, Nordic European Molecular Biology Laboratory Partnership for Molecular Medicine, Aarhus University, 8000 Aarhus C, Denmark

[1]M.M.G.G., J.D.C., and B.A. contributed equally to this work

[2]To whom correspondence may be addressed. **Email:** maike.bublitz@bioch.ox.ac.uk

This PDF file includes:

Figures S1 to S5
Tables S1 to S2
Legends for Movies S1 to S2

Other supplementary materials for this manuscript include the following:

Movies S1 to S2

www.pnas.org/cgi/doi/10.1073/pnas.2014896117

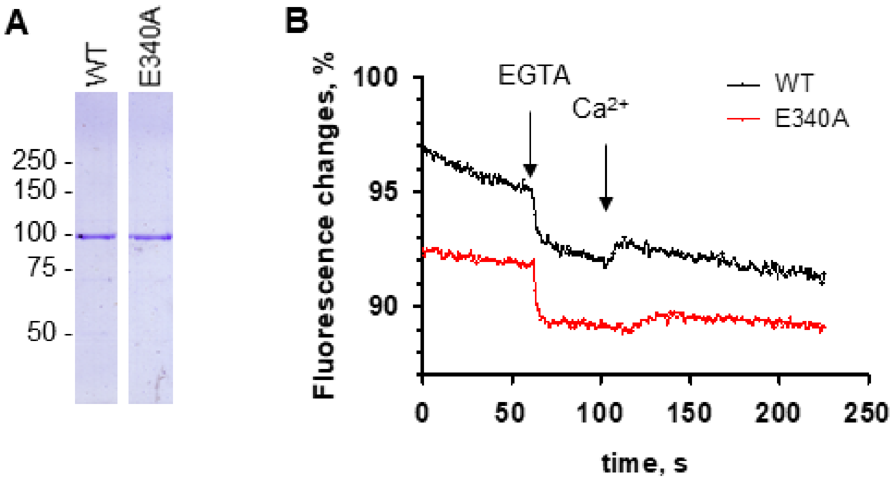

Figure S1: Gel electrophoresis and tryptophan fluorescence measurements.

A Coomassie blue stained SDS-PAGE of the purified SERCA WT (WT) and E340A proteins.

B SERCA1a intrinsic fluorescence changes measured with purified WT (black) or E340A (red) starting at 105 µM Ca^{2+} followed by addition of 5 mM EGTA and 12.5 mM extra Ca^{2+}. Fluorescence intensities are given as a percentage of the fluorescence level at start. For sake of clarity, data for E340A were shifted down manually by 10%. The traces shown are the average of 4 experiments for WT and 3 experiments for E340A.

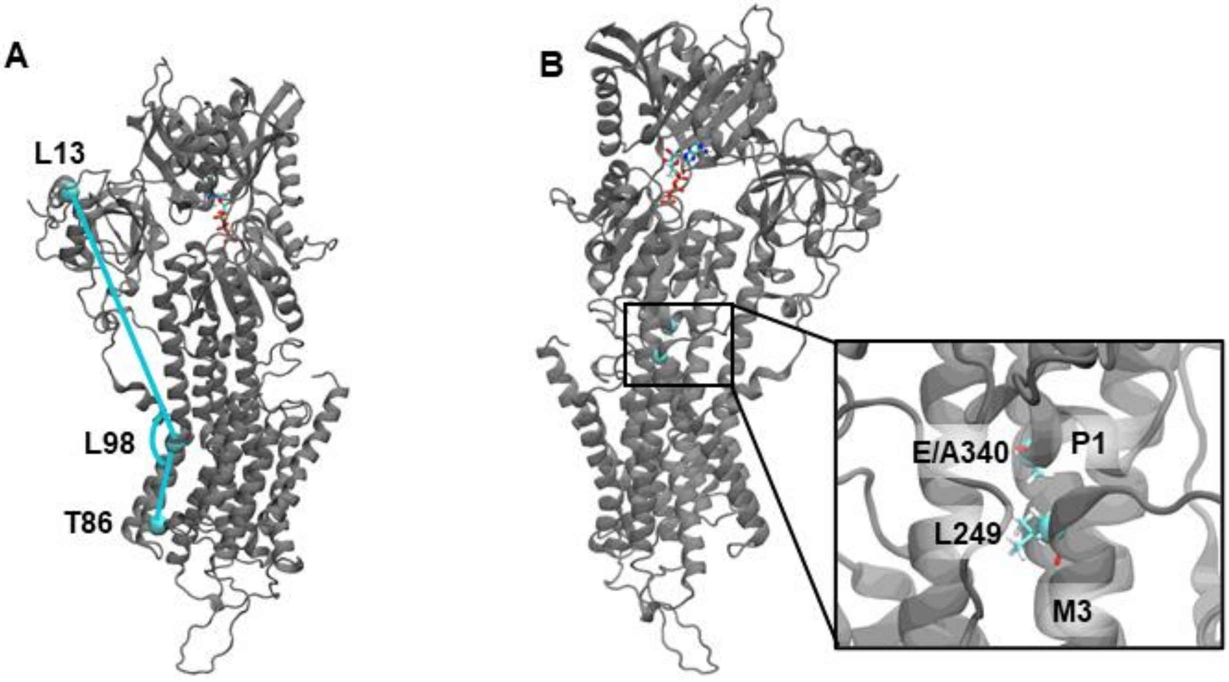

Figure S2: Visualization of MD simulation analyses of E340A conformation.

A Residues used to determine the headpiece angle, Leu13, Thr86 and Leu98.

B Location of residues Glu/Ala340 on P1 and Leu249 in M3.

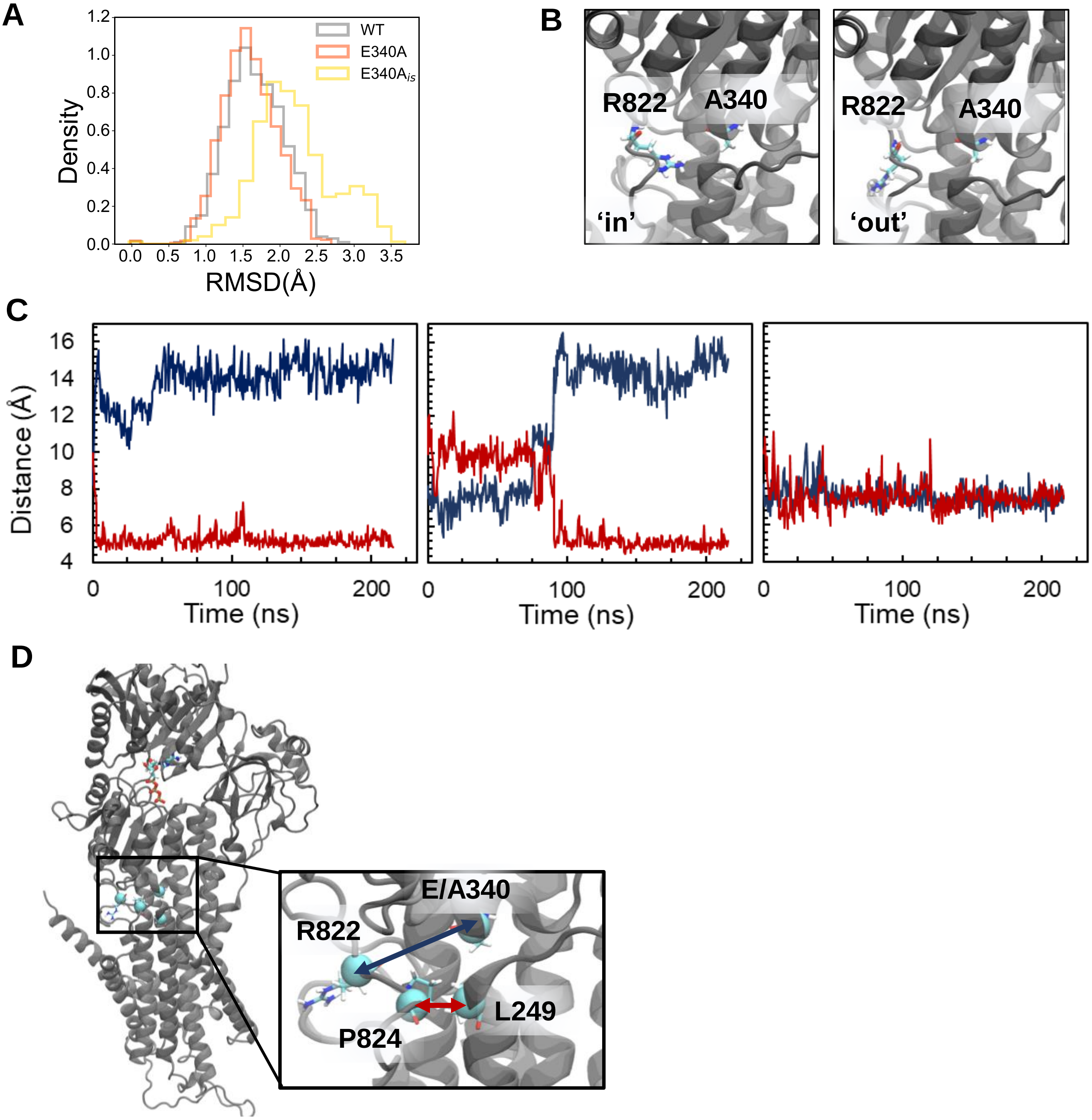

Figure S3: MD simulation analysis of the L6-7 loop.

A RMSD histogram of L6-7 over the course of the simulation.

B 'In' (left) and 'out' (right) positions of Arg822.

C Distance traces between Arg822-Nε and Glu/Ala340-Cα (blue) and between Leu249-Cα and Pro824-Cα (red) in E340A$_{is}$. Left to right: simulation runs 1-3.

D Visualization of the distance measurements between Glu/Ala340 and Arg822 and between Leu249 and Pro824.

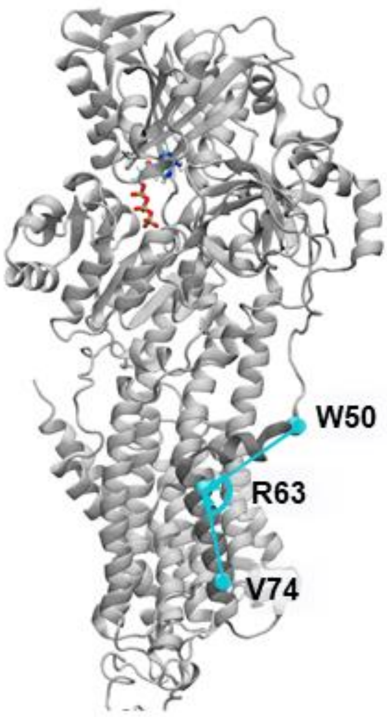

Figure S4: Visualization of MD analysis of the M1 kink, measured between residues Trp50, Arg63 and Val74.

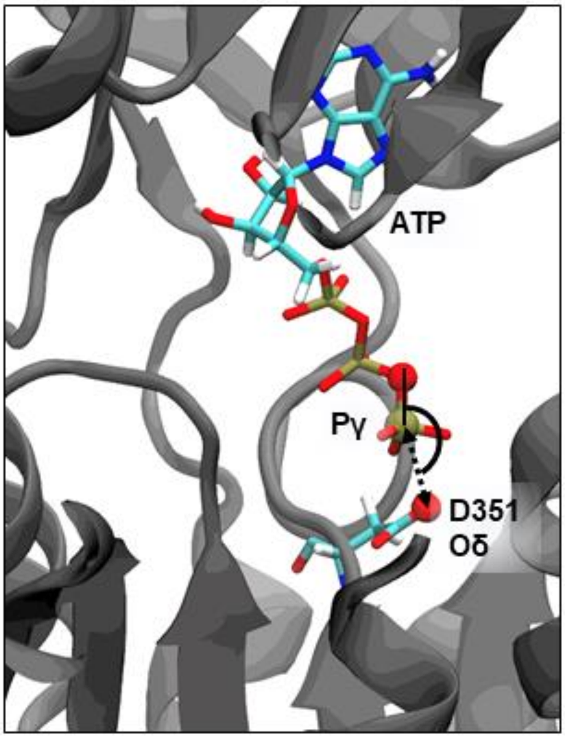

Figure S5: Visualization of MD analysis of the phosphorylation site geometry.

Table S1: SERCA Glu340 is conserved in ion- and lipid transporting P-type ATPases.
In the P-type ATPase database (http://www.traplabs.dk/patbase/Motifs.html), the glutamate is conserved in 135 out of 159 P-type ATPases. The P3B and P5 subfamilies are the only groups where it systematically misses, being replaced by N and Q. In P1B, a few bacterial pumps have Q, S, or D.

P-type Family	Protein	Substrate	Organism	P1 Sequence
P1A	KdpB	K^+	*E. coli*	RAVEAAGD
P1B	ATP7A	Cu^+	*H. sapiens*	EPLEMAHK
P2A	SERCA1A	Ca^{2+}	*H. sapiens*	PSVETLGC
**P2A**	**SERCA1A**	**Ca^{2+}**	***O. cuniculus***	PSVETLGC
P2B	PMCA	Ca^{2+}	*H. sapiens*	DACETMGN
P2C	Na^+,K^+-ATPase	Na^+, K^+	*H. sapiens*	EAVETLGS
P2C	H^+,K^+-ATPase	H^+, K^+	*H. sapiens*	EAVETLGS
P2D	CTA3	Ca^{2+}	*S. pombe*	EALEALGG
P3A	AHA2	H^+	*A. thaliana*	TAIEEMAG
P3A	Pma1	H^+	*N. crassa*	SAIESLAG
P3B	MgtA	Mg^{2+}	*E. coli*	DAIQNFGA
P4	ATP8B1	Phospholipid	*H. sapiens*	TLNEQLGQ
P5	ATP13A2	Polyamine	*H. sapiens*	QRINVCGQ

Table S2: Crystallographic data collections and refinement statistics. Values in parentheses refer to the highest resolution shell

**Data collection**	
Beamline	ESRF ID23-2
Space group	$P2_12_12$
Unit cell (Å, °)	a=232.90, b=126.99, c=49.81, α=β=γ=90
Wavelength (Å)	0.8729
Resolution (Å)	75-3.2 (3.3-3.2)
Number of unique reflections	25337 (2201)
Completeness (%)	99.9 (99.8)
Multiplicity	6.1 (6.2)
I/σI	10.9 (1.3)
Rmeas	0.196 (>100)
$CC_{1/2}$ in highest resolution shell	0.47
Wilson B-factor	97.8
**Refinement**	
Resolution (Å)	49.81-3.2
Rwork/Rfree (%)	0.21/0.26
Rmsd bond (Å)	0.009
Rmsd angle (°)	0.691
Mean B-factors ($Å^2$)	90.7
Ramachandran plot (%) Favoured/allowed/outliers	93.0/6.4/0.6

Movie S1 (separate file). Morph between equivalent Ca_2E1-AMPPCP crystal structures of SERCA WT (PDB 3N8G) and E340A (PDB 6RB2). The inset is a zoom on the region around residue 340 (green sphere).

Movie S2 (separate file). Superposed morphs between crystal structures of Ca^{2+}-free E1 WT (PDB 4H1W) and Ca_2E1-AMPPCP forms of WT (PDB 3N8G, light colors) and E340A (PDB 6RB2, dark colors). The inset is a zoom on the region around residue 340 (green sphere).
